# Correspondence

**Published:** 1864-01

**Authors:** B. Wood

**Affiliations:** Albany


### Correspondence.

ALBANY, Dec. 29, 1863.

Prof. J. TAFT: *Dear Sir:*—In submitting the enclosed communication, I do not see how, under the circumstances, I am to avoid accompanying it with some explanatory remarks. I am aware that, in consequence of not being able to devote the necessary attention to other subjects with which I was less acquainted, I proposed something of the kind (in fact the most *useful* thing for the profession I could offer), and that your reply was—send it on. Yet I have been disposed to put it off until the last, thinking something else might turn up, when the "spirit moved" to write. I will now let the impulse take its course.

It is really a pleasure to contribute to your valuable journal anything you may deem worth publishing, and a source of gratification to hold occasional communication through it with members of our profession.

But the subject in hand relates to an article which the writer manufactures and offers for sale, and, indeed, advertises in another place; and any further reference to it may but serve as an additional advertisement possibly indirectly aiding said sale, thereby contributing to his pecuniary benefit. Now the question arises (and I can not here ignore it), whether, and how far, such consideration should operate to the exclusion or acceptance of matter that might otherwise be esteemed of sufficient general interest to warrant its publication? And how far should it restrain one having a due sense of delicacy, self-respect and deference toward his compeers, in offering anything of the kind as a communication? This question would seem to require preliminary solution.

But presuming that otherwise acceptible matter ought not to be excluded merely on such ground, and may therefore be offered without inpropriety, although the question is but sprung, and far from being decided, if indeed it be not decided adversely; yet it is due to add a word or two apologetic, or at least explanatory, in the present case.

Although brief directions for using the material go with it, and thus fall into the hands of some readers of the journals, members of the profession had suggested the need of fuller and more explicit information as to the manner of using it, and I found it was expected of me to make such communication through the Dental journals, the *Cosmos* being generally designated, as presumed to have the largest circulation in this section. It seemed right on my part to meet these expectations; and not to do so, in such public manner, might subject me to the charge of purposely withholding this information from all but purchasers. There seemed to be no impropriety in submitting it, nor ground for objection to affording it wider circulation. What harm, even, to give general publicity to what was already in print but confined to a few? Suppose some European dentist had discovered, or professed to have discovered something which promised to be of advantage to the profession, would any journal sincerely devoted to the interests of that profession be backward to communicate whatever information might be accessible upon the subject?

Besides, information as to the manner of using a thing furnishes the best clue to a practical understanding of its nature. If seen to involve trouble in the using, some might thereby be spared the expense of purchasing. And how many, engaged in dental practice, look and care for nothing but what involves the least possible trouble, care and responsibility in the using! Now, for my own part, however much pecuniary benefit be an object of desire or need, I want no one's money unless I can offer an equivalent; and that which he can not use, or will not trouble himself to learn the use of, is no equivalent to *him*, however valuable it may be in the hands of others.

It was with reflections something like these, that I did draw up an article on the subject and submitted it to the journal designated, which it is due to you, to state, as it is substantially the same as that herewith submitted for the Register; as well as that a synopsis of it has since been printed, and, to a limited extent, circulated. It also becomes me to state frankly, as it may influence your own action, that after due consideration, it was regarded by both the editor and proprietor of that journal as something which "belonged properly to the advertising pages," and was accordingly declined "as a communication," in conformity with a rule adopted by that journal under which "communications received in reference to improvements made by the authors—are usually declined as communications when the improvements to which they relate are offered for sale to the profession,—a rule which applies" to the proprietor "as well as to other parties."

With this statement I leave the article at your disposal. If you publish it notwithstanding, it will be proper to accompany it with this explanation, so that no misconception shall arise. I send it just as it is, not thinking it worth while to make any amendations in matter or style; for, since neither the matter, in itself, nor the style was objected to, it would be just as improper in me to offer you an entirely new article upon *this subject* as the identical one rejected by a contemporary on the grounds given. I do not consider the grounds valid (although I do not say it to influence the case), but propose, in a subsequent number, to show somewhat at length wherein I conceive this principle of adjudication to be detrimental, not to this or that man's pecuniary benefit, but to the great interests of the profession at large.

Yours, very respectfully, B. Wood.

